# Inflammatory markers in cerebrospinal fluid in pediatric Brazilian spotted fever with neurological manifestations

**DOI:** 10.1590/S1678-9946202567032

**Published:** 2025-05-26

**Authors:** Daniela Caldas Teixeira, Pedro Alves Soares Vaz de Castro, Maria Clara de Araujo Gontijo Lima, Roberta Maia de Castro Romanelli, Ana Cristina Simões e Silva, Lilian Martins Oliveira Diniz

**Affiliations:** 1 Universidade Federal de Minas Gerais, Faculdade de Medicina, Departamento de Pediatria, Belo Horizonte, Minas Gerais, Brazil; 2 Secretaria de Estado de Saúde de Minas Gerais, Belo Horizonte, Minas Gerais, Brazil; 3 Universidade Federal de Minas Gerais, Faculdade de Medicina, Belo Horizonte, Minas Gerais, Brazil; 4 Fundação Hospitalar de Minas Gerais, Hospital Infantil João Paulo II, Belo Horizonte, Minas Gerais, Brazil

Belo Horizonte, March 4^th^, 2025

Dear Editor,

Rocky Mountain spotted fever is a severe infectious disease caused by bacteria of the *Rickettsia* genus, which is primarily transmitted by ticks. In Brazil, it is known as Brazilian spotted fever (BSF) and *Rickettsia rickettsii* is the most common etiological agent. The disease have a broad clinical spectrum, ranging from nonspecific febrile illnesses to severe multisystem dysfunction and is associated with high mortality rates when diagnosis and treatment are delayed^1^.

Although neurological manifestations are less common, they may occur, particularly in severe cases. The most frequently reported neurological manifestations in rickettsial infections include meningitis, encephalitis, and acute disseminated encephalomyelitis. However, other conditions, such as unilateral facial nerve paralysis, cerebral infarction, vision loss, and intracranial hypertension, have also been reported^2–5^. Moreover, severe sequelae have been described in survivors, including hemiplegia, hearing loss, visual impairments, slurred speech, mental confusion, and cranial neuropathies, which can persist for weeks after recovery^2^.

The precise mechanisms underlying rickettsial pathogenesis in the central nervous system (CNS) remain only partially understood. It is suggested that neurological manifestations result from an exaggerated inflammatory response and endothelial dysfunction, hallmarks of the disease, leading to microvascular damage and disruption of biological barriers such as the blood-brain barrier^2,6,7^. The understanding of the immunological mechanisms involved in these manifestations is limited, particularly in pediatric patients, whose immune responses differ from adult individuals.

In this context, this study delves deeper into a previously reported case^8^, aiming to characterize the inflammatory profile of a pediatric patient with confirmed BSF, by analyzing cytokines and chemokines in cerebrospinal fluid, and to correlate such findings with CNS involvement.

A 20-month-old male child from a rural area went to the emergency department with a six-day history of fever and a rash involving the trunk, limbs, and face. Four days after symptoms onset, the child developed neurological manifestations, including loss of ambulation and speech. The mother reported finding a tick on the child 15 days prior to the onset of symptoms.

On admission to the referral center, the child had fluctuating sensorium, poor eye contact, aphasia, axial hypotonia, left hemiparesis, neck stiffness, and a positive Brudzinski's sign. The CSF analysis showed mild pleocytosis (80 cells/mm^3^) with lymphocyte predominance (88%), hypoglycorrhachia (43 mg/dL), and elevated protein levels (95 g/L). The cranial computed tomography was normal. An empiric treatment was initiated on the sixth day of symptoms with intravenous ceftriaxone and oral doxycycline, based on clinical suspicion of bacterial meningitis and BSF.

A PCR test for *Rickettsia* spp., which was performed on a blood sample, yielded positive, confirming the diagnosis of BSF. Serologic tests for BSF were initially negative but became positive on the follow-up, confirming seroconversion. The patient's neurological symptoms gradually resolved, and by the eighth day of treatment, he was able to walk and talk again. After 10 days of doxycycline therapy, the child was discharged with full neurological recovery.

An aliquot of CSF collected at admission was stored at −20 °C and allocated for the evaluation of inflammatory markers. The simultaneous quantification of IL-1β, IL-6, IL-8, IL-10, TNF, and IL-12p70 was performed using the CBA Human Inflammatory Cytokine Kit (BD Biosciences, San Jose, CA, USA) by flow cytometry (BD FACSCanto™II, BD Biosciences, San Jose, CA, USA). The results were compared to those of a male pediatric patient of similar age treated at the same institution who had confirmed *Streptococcus pneumoniae* bacterial meningitis, as well as to an experimental negative control (0 pg/mL). Both patients were enrolled in a research project investigating cytokine and chemokine profiles in CSF from CNS infections, which was approved by the Research and Education Committees of the Fundacao Hospitalar do Estado de Minas Gerais and the Universidade Federal de Minas Gerais under ethics approval N° CAAE 21781914.2.0000.5119.

**Figure 1:**
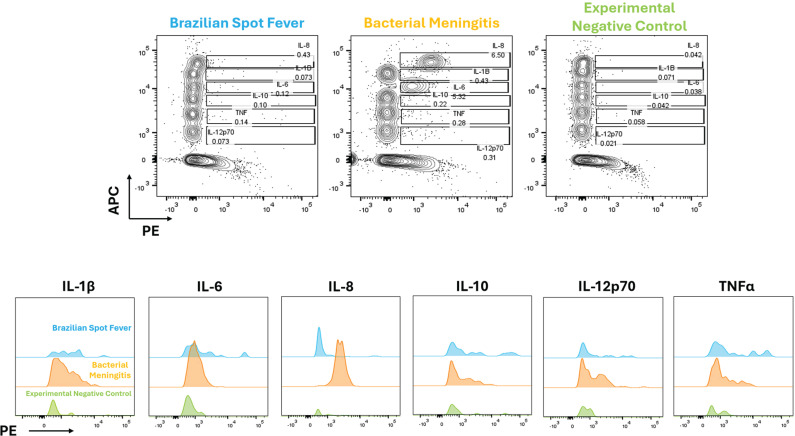
Cytokine profile in cerebrospinal fluid of a pediatric patient with Rocky Mountain spotted fever compared to bacterial meningitis and negative control.

Figure 1 shows that the patient with BSF had elevated levels of IL-6 (90.46 pg/mL) and TNF-α (1.12 pg/mL) compared to the negative control (undetectable), although these levels were markedly lower than those observed in the patient who had bacterial meningitis (IL-6: 2015.44 pg/mL, TNF-α: 9.65 pg/mL). Similarly, IL-1β (239.62 pg/mL), IL-8 (144.37 pg/mL), IL-10 (0.67 pg/mL), and IL-12p70 (5.24 pg/mL) were moderately elevated in the BSF patient but significantly higher in the bacterial meningitis case (IL-1β: 428.09 pg/mL, IL-8: 3651.44 pg/mL, IL-10: 1.02 pg/mL and IL-12p70: 21.61pg/mL). All cytokines exceeded the levels observed in the negative control (undetectable). Such findings underscore the distinct inflammatory activation in BSF, which, although less intense than the robust immune response observed in bacterial meningitis, is characterized by a unique cytokine profile.

The differences in the intensity and pattern of the inflammatory response between BSF and bacterial meningitis can be attributed to factors such as pathogen characteristics, infection tropism, and host immune interactions. *Rickettsia rickettsii*, the causative agent of BSF, is an obligate intracellular bacterium that primarily infects endothelial cells, leading to a vascular injury and triggering a localized immune response. This response is characterized by the production of pro-inflammatory cytokines, including IL-6 and TNF-α, which contribute to blood-brain barrier dysfunction and subsequent neurological changes^4,9^. In contrast, bacterial meningitis pathogens such as *Neisseria meningitidis* and *Streptococcus pneumoniae* are extracellular organisms capable of rapidly invading the CSF. Such invasion provokes a robust and widespread inflammatory response, mediated predominantly by neutrophils and marked by elevated levels of IL-1β and IL-8, which play a key role in leukocyte recruitment and activation^10,11^.

The site of infection also influences the immune response. In BSF, the damage is primarily vascular, which leads to focal inflammation, while in bacterial meningitis, direct infection of the CSF and meninges results in a more intense and diffuse immune activation. Furthermore, it is typical for BSF to gradually progress more in its early stages, enabling the immune response to remain localized to the endothelial infection before spreading^4,9^. Conversely, bacterial meningitis often progresses rapidly, with overwhelming activation of innate immunity in the CNS.

Cytokine modulation also differs between these conditions. For instance, IL-10, an anti-inflammatory cytokine, may play a more pronounced regulatory role in BSF, potentially limiting the inflammatory response. An imbalance between pro and anti-inflammatory cytokines often leads to an unregulated inflammatory response in bacterial meningitis, exacerbating tissue damage^10–12^. Such distinctions highlight not only the biological characteristics of the pathogens but also the complex dynamics of host immune responses to different infection sites and types.

There is a lack of studies establishing precise reference values for specific cytokines in the CSF of healthy children, because most research focus on pathological contexts, such as central nervous system infections or inflammatory diseases. Reference values also vary depending on the detection methods and population characteristics. This study is limited by the evaluation of a single patient and comparisons with control cases, which cannot be considered definitive evidence. Additionally, factors such as individual burden of disease and immune response must be accounted for. However, to the best of our knowledge, this is the first description of an inflammatory profile in the CSF of a patient with neurological manifestations of BSF. Such findings provide a basis for future research and offer insights into the pathophysiological mechanisms of this study.
